# 3PO inhibits inflammatory NFκB and stress-activated kinase signaling in primary human endothelial cells independently of its target PFKFB3

**DOI:** 10.1371/journal.pone.0229395

**Published:** 2020-03-04

**Authors:** Jonas Aakre Wik, Peter Lundbäck, Lars la Cour Poulsen, Guttorm Haraldsen, Bjørn Steen Skålhegg, Johanna Hol

**Affiliations:** 1 Department of Pathology, Oslo University Hospital-Rikshospitalet, Oslo, Norway; 2 Department of Pathology, Institute of Clinical Medicine, University of Oslo, Oslo, Norway; 3 K.G Jebsen Inflammation Research Centre, Faculty of Medicine, Institute of Clinical Medicine, University of Oslo, Oslo, Norway; 4 Department of Nutrition, Division of Molecular Nutrition, Institute of Basic Medical Sciences, University of Oslo, Oslo, Norway; Medical College of Georgia at Augusta University, UNITED STATES

## Abstract

Inhibition of the key glycolytic activator 6-phosphofructokinase 2/fructose-2,6-bisphosphatase-3 (PFKFB3) by 3-(3-pyridinyl)-1-(4-pyridinyl)-2-propen-1-one (3PO) strongly attenuates pathological angiogenesis in cancer and inflammation. In addition to modulating endothelial proliferation and migration, 3PO also dampens proinflammatory activation of endothelial cells and experimental inflammation *in vivo*, suggesting a potential for 3PO in the treatment of chronic inflammation. The aim of our study was to explore if the anti-inflammatory action of 3PO in human endothelial cells was mediated by inhibition of PFKFB3 and glycolysis and assess if other means of PFKFB3 inhibition reduced inflammatory activation in a similar manner. We found that 3PO caused a rapid and transient reduction in IL-1β- and TNF-induced phosphorylation of both IKKα/β and JNK, thus inhibiting signaling through the NFκB and the stress-activated kinase pathways. However, in contrast to 3PO-treatment, neither shRNA-mediated silencing of PFKFB3 nor treatment with the alternative PFKFB3 inhibitor 7,8-dihydroxy-3-(4-hydroxy-phenyl)-chromen-4-one (YN1) prevented cytokine-induced NFκB signaling and upregulation of the adhesion molecules VCAM-1 and E-selectin, implying off target effects of 3PO. Collectively, our results suggest that the anti-inflammatory action of 3PO in human endothelial cells is not limited to inhibition of PFKFB3 and cellular glycolysis.

## Introduction

Cellular metabolism is closely linked to cellular activation states and input from the environment [1]. Within the microenvironment of inflammatory lesions, cells are exposed to hypoxia, pro-inflammatory cytokines, angiogenic mediators, and pathogen-associated molecules, all cues that serve to increase the rate of glycolysis in a range of cell types, including endothelial cells [1, 2]. To this end, strategies that target cellular glycolysis have been proposed as a potential therapeutic approach to ameliorate inflammation [1].

Phosphofructokinase-2/fructose-2,6-bisphosphatase-3 (PFKFB3) is an activator of the key glycolytic enzyme 6-phosphofructokinase-1 (PFK-1), responsible for converting fructose-6-phosphate to fructose-1,6-bisphosphate. PFKFB isoenzymes synthesize fructose-2,6-bisphosphate, an allosteric activator of PFK-1 that potently stimulates glycolysis [1, 3]. In addition to activating PFK-1, fructose 2,6-bisphosphate regulates the activity of the cyclin-dependent kinase 1 [4], and, in line with this, PFKFB3 is transiently upregulated in mitotic cells at the nutrient sensitive check point (G_1_-S) [4].

PFKFB3 has been considered a promising therapeutic target in cancer, primarily because of its role in supporting the high glycolytic rates required to fuel rapid cellular proliferation [5]. In addition, studies based on genetic inhibition revealed that PFKFB3 is also a strong driver of proliferation and migration in angiogenic endothelial cells [2, 6]. Accordingly, partial inhibition of the enzyme dampened dysregulated angiogenesis and restored vascular integrity in solid tumors, with the consequence of improving the delivery of chemotherapeutic agents and reducing metastasis [4, 7]. The interest in PFKFB3 as a therapeutic target led to the development of several small molecules that inhibit PFKFB3 enzyme activity, including 3-(3-pyridinyl)-1-(4-pyridinyl)-2-propen-1-one (3PO) and 7,8-dihydroxy-3-(4hydroxyphenyl) chromen-4-one (YN1) [8, 9]. Indeed, inhibition of PFKFB3 by 3PO and YN1 resembled the effects of genetic inhibition of PFKFB3, by reducing angiogenic sprouting *in vitro* and *in vivo* [10], and, in support of their mode of action, reducing the rate of glycolysis in primary human umbilical cord vein endothelial cells (HUVECs) [10].

The use of 3PO was expanded to assess efficacy in experimental inflammation, revealing that repeated injections ameliorated pathological angiogenesis and reduced leukocyte infiltration in mouse dextran sulfate sodium-induced colitis and imiquimod-induced psoriasis-like dermatitis [10]. Moreover, 3PO also increased the survival of mice exposed to experimental sepsis, by alleviating pulmonary inflammation [11].

At the cellular level, 3PO modulated IL-1β-induced nuclear factor kappa B (NFκB) signaling and adhesion molecule upregulation in cytokine-stimulated endothelial cells, by interfering with early activation of the NFκB signaling cascade as measured by phosphorylation and degradation of the inhibitory molecule IκBα [7]. Likewise, 3PO also reduced the hypoxia-induced inflammatory activation of macrophages [12] and rheumatoid arthritis synovial fibroblasts [13, 14].

The molecular mechanisms by which PFKFB3 controls endothelial cell migration rely at least partly on localization of the enzyme to membrane protrusions, where it contributes to the glycolytic machinery that is required for rapid actin polymerization and lamellipodia formation [2]. Furthermore, the effect of PFKFB3 on cell cycle progression is mediated both by increasing cellular ATP and by directly regulating the activity and expression levels of cyclin kinases and other regulatory proteins [4, 15, 16]. In contrast, the molecular mechanism by which 3PO interferes with inflammatory signaling remains unknown.

Notably, 3PO has a narrow therapeutic index, and the dose that mediates vascular normalization and improves barrier function is only marginally lower than doses that cause toxicity and barrier disruption [17].

Here, we have investigated whether interference with inflammatory signalling in endothelial cells by 3PO is solely due to inhibition of PFKFB3 and glycolysis. We found that 3PO transiently inhibited phosphorylation of both IKKα/β- and JNK after stimulation with IL-1β and TNF. However, both genetic ablation of PFKFB3 and pharmacological inhibition of PFKFB3 using YN1 failed to reproduce 3PO-mediated effects on cytokine-induced expression of adhesion molecules.

Our results suggest that the effect of 3PO has off target effects and inhibits inflammatory activation of human endothelial cells independent of PFKFB3.

## Materials and methods

### Reagents

IL-1β, TNF, EGF, bFGF and VEGF 165 were purchased from R&D systems. Hydrocortisone, YN1, and DMEM without glucose, L-glutamine, sodium pyruvate powder were purchased from Sigma Aldrich. Fetal calf serum (FCS), gentamicin, fungizone, L-glutamine, MCBD 131, Opti-MEM and TRI-reagent were purchased from Thermo Fisher Scientific. Trypsin and EDTA were purchased from BioWhittaker. 3PO was purchased from Merck Millipore and dissolved in DMSO. HEPES buffer, Rotenone, Antimycin A, and 2-deoxyglucose were purchased from Agilent in the glycolytic rate assay starter kit.

### Cell culture

HUVECs were isolated as described [18]. Cells were grown on 0.1% gelatin coated plastic, cultured in a 5% CO_2_ 95% humidity incubator at 37°C, and used in passage 2–5. Cells were grown in MCDB 131 medium supplemented with 7.5% FCS, 2mM L-glutamine, 10 ng/ml rh-EGF, 1 ng/ml rh-bFGF, 1 μg/ml hydrocortisone, 50 μg/ml gentamicin and 250 ng/ml fungizone for culture expansion. In preparation for experiments, cells were grown in MCDB with 2% FCS, 2 mM L-glutamine, 5 ng/ml rh-EGF, 10 ng/ml rh-bFGF, 20 ng/ml Long R3 IGF, 0.5 ng/ml rh-VEGF 165, 1 μg/ml ascorbic acid, 0.2 μg/ml heparin 50 μg/ml gentamicin, and 250 ng/ml fungizone. Interleukin 1β (IL-1β) and tumor necrosis factor (TNF) was purchased from R&D systems and used at a concentration of 1 ng/ml.

### Western blot

Cells were washed with ice cold PBS, then lysed in 10 mM Tris buffer pH 6.8, containing 5mM EDTA, 6 mM sodium fluoride, 5 mM tetrasodium pyrophosphate, 2% SDS, protease inhibitors (Sigma P5726, 1:100) and phosphatase inhibitors (Sigma P8340, 1:100). Sample buffer (72% glycerol, 28% β-mercaptoethanol, 0.33 mg/ml bromophenol blue) was added at a 1:7 ratio (v/v). Samples were heated to 65°C for 10 minutes. Approximately 10 μg protein was loaded to each well on 15 well 10% or 4–20% BioRad mini-PROTEAN®TGX™ precast gels. Proteins were transferred to a nitrocellulose membrane using the Trans-blot® Turbo™ transfer system using the program ‘mixed MW’. All further steps were performed at room temperature with agitation unless otherwise stated: Membranes were blocked with 5% no-fat milk (BioRad) in TBST (Tris-buffered saline with 0.01% Tween 20, pH 7.4) or 5% BSA in TBST for 30 minutes, incubated with antibodies diluted in either of these buffers overnight (4°C), washed with TBST and finally incubated with appropriate HRP-conjugated secondary antibodies for 2 hours. Substrate (SuperSignal™ West Dura Extended Duration Substrate, Thermo Fisher Scientific) was added to the membrane and signal was detected using ChemiDoc XRS+ system and Image Lab 4.1. When necessary, membranes were stripped using Restore PLUS western blot stripping buffer (Thermo Fisher Scientific) for 10 minutes, then washed with PBS three times before membranes were blocked and stained with new antibodies. Bands were quantified using volume tools in Image Lab 4.1 and normalized to β-tubulin. Phospho-proteins were normalized to unphosphorylated protein, then to β-tubulin. Antibodies against phosphorylated IKKα/β (Ser176/180) (mAb clone 16A6, 1:1000 in 5% BSA), IKKβ (mAb clone D30C6, 1:1000 in BSA), phosphorylated SAPK/JNK (Thr183/Tyr185) (JNK) (polyclonal #9251, 1:1000 5% BSA) and SAPK/JNK (JNK) (polyclonal #9252 1:1000, 5% BSA) were purchased from Cell Signaling Technologies, anti-Vascular cell adhesion molecule-1 (VCAM-1) (polyclonal BBA19, 1:1000 1% milk), was purchased from R&D Systems, anti-PFKFB3 (mAb clone EPR12594, 1:2000 1% milk) and anti-β-tubulin (polyclonal ab6046, 1:20000, 1% milk) were purchased from Abcam. HRP-conjugated anti-goat IgG (polyclonal sc-2020, 1:10000, 1% milk) was purchased from Santa Cruz Biotechnology. HRP-conjugated anti rabbit IgG (polyclonal 711-035-153, 1:20000, 1% milk) was purchased form Jackson ImmunoResearch.

### Reverse transcriptase quantitative PCR

Cells were washed in PBS at room temperature and lysed in Tri-Reagent. RNA was extracted using 1-bromo-3-chloropropane, isopropanol and ethanol extraction, dissolving RNA in DEPC H_2_O. cDNA was synthesized using SuperScript III® reverse transcriptase according to manufacturer’s instructions. Primers for *VCAM1* (AGT TGA AGG ATG CGG GAG TAT, GGA TGC AAA ATA GAG CAC GAG 2.0 mM mg/Cl_2_), *PFKFB3* (GTC CCT TCT TTG CAT CCT CTG, CCT ACC TGA AAT GCC CTC TTC 1.5 mM MgCl_2_), *SELE*, and *HPRT* (AAT ACA AAG CCT AAG ATG AGA GTT CAA GTT GAG TT, CTA TAG GCT CAT AGT GCA AAT AAA CAG TTT AGG AAT, 2.0 mM MgCl_2_) were designed to span exon-exon junctions using Primer3 and BLAST. qPCR was performed using the AriaMX Real-time PCR system (Agilent). Relative quantities were calculated using the ΔΔCT method, using *HPRT* as the housekeeping gene.

### Metabolic assay

Cells were seeded at a density of 4x10^4^ cells/well in gelatin-coated Seahorse XF24 polystyrene cell culture plates on the day before performing the assay. DMEM without NaHCO_3_ or phenol red supplemented with 2 mM L-glutamine, 10 mM D-glucose, 1 mM sodium pyruvate and 5 mM HEPES buffer adjusted to pH 7.4 was used as assay medium. Cells were washed 3 times with this medium 1 hour prior to running the assay and incubated in a humidified non-CO_2_ incubator at 37°C according to the manufacturer’s instructions. Extracellular acidification rate (ECAR) and oxygen consumption rate (OCR) were measured in 5 cycles between each injection (2 minutes mixing, 2 minutes recovery and 2 minutes measurement), and the respective rates were determined. This was done using the company protocol for the glycolytic rate assay kit (Agilent) by injections of equimolar concentration of rotenone and antimycin A (Rot/AA) (5 μM) followed by injection of 2-deoxy-glucose (2DG) (50 mM). Measurements were normalized to cell density by fixing cells in 0.5% paraformaldehyde-lysine-periodate in PBS then adding 120 μl 0.1% crystal violet in PBS to each well incubated at room temperature for 3–4 minutes followed by washing with tap water. Crystal violet was solubilized by adding 33% acetic acid and OD was measured 550 nm using a microplate reader.

### Cell ELISA

Relative protein expression of VCAM-1 and E-selectin was estimated by ELISA in fixed HUVECs as described [19]. Cells were seeded in gelatin-coated 96 well plates at a density of 4 x10^4^ cells/cm^2^ and cultured for 24 hours before stimulation with IL-1β (1ng/ml) in the presence or absence of 3PO (20 μM, IC50 = 22.9 μM) or YN1 (20 μM, IC50 0.67 μM) for 3 hours. Cells were fixed in in 0.5% paraformaldehyde-lysine-periodate in PBS for 10 minutes at room temperature before air-drying overnight at 4°C. Cells were permeabilized with 0.1% saponin for 5 minutes followed by incubation with antibodies against VCAM-1 (clone 4B9, John Harland, Seattle, 1 μg/ml) or E-selectin (BBA16, 1 μg/ml) for 45 minutes. The plate was washed with PBS 3 times and incubated with HRP-conjugated anti-mouse IgG (Sigma, A3673) for 45 minutes, then washed 3 times with PBS. KPL TMB peroxidase Substrate was prepared as described by manufacturer and 100 μl was added to all the wells. Reaction was stopped by adding 100 μl 1 M H_2_SO_4_ per well. Optical densities were measured at 450 nm using plate reader. To determine relative cell density, plates were washed in cold tap water 5 times, then 70 μl 0.1% crystal violet (Ås Produksjonslab AS, Norway) in PBS was added to each well and incubated at room temperature for 4 minutes, then washed 5 times in cold tap water. Crystal violet stain was dissolved in 100 μl 33% acetic acid and OD was measured at 550 nm by plate reader. Absorbance values for VCAM-1 and E-selectin was divided by the OD values for crystal violet to normalize against cell density.

### Lentiviral transduction

Cells were seeded at density of 3x10^4^ cells/cm^2^ in gelatin-coated T25 polystyrene cell culture flasks, and cultured for 4 hours, then transduced by lentivirus-expressing short hairpin RNA (shRNA) sequence matching PFKFB3 (shPFKFB3) or an empty vector (Empty). Cells were cultured for 2 days, then seeded for experiments.

### Transfection and knockdown of PFKFB3

Cells were seeded at a density of 4x10^4^ cells/cm^2^ in gelatin coated 12 well polystyrene plates then cultured for 24 hours. Prior to transfection medium was changed to growth medium without fungizone and gentamicin. Silencing RNA (siRNA) (27 nM) was mixed with Lipofectamine RNAiMAX according to manufacturer’s instructions (Thermo Fisher Scientific). Medium was changed to growth medium with fungizone and gentamicin 6 hours later. Cells were cultured for 48 hours then used for experiments. Pre-designed non-targeting siRNA (Scramble) and siRNA targeting PFKFB3 (siPFKFB3) were obtained from Thermo Fisher Scientific (Silencer Select).

### Statistical methods

Statistical analyses were performed using student’s T-test for normally distributed data and Wilcoxon signed-rank test for qPCR data normalized to internal control using GraphPad Prism (Version 8.1.2).

### Ethics statement

Human umbilical cords were collected after written informed consent by a protocol approved by the Regional Committee for Medical Research Ethics (2014/298 S-05152a), Health Region South, Norway.

## Results

### 3PO delays IL-1β- and TNF-induced phosphorylation of IKKα/β and JNK

**Fig 1:**
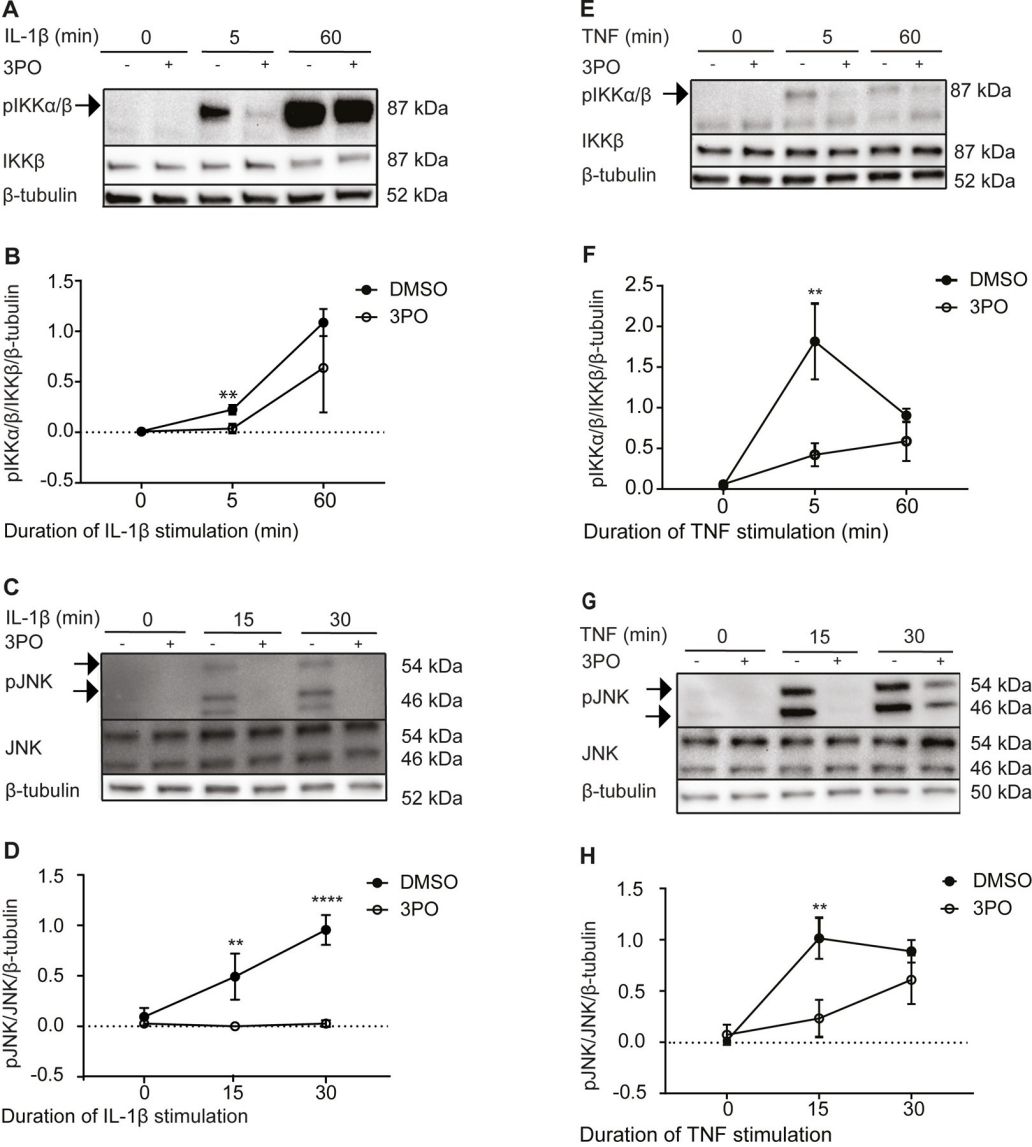
3PO treatment inhibits IL-1β- and TNF-induced phosphorylation of IKKα/β and JNK in endothelial cells. (A) Representative immunoblots of HUVEC stimulated with IL-1β (1 ng/ml) for 0, 5, and 60 minutes in the presence (+) or absence (-) of 3PO (20 μM) pretreatment for 30 minutes. Panels show phosphorylated IKKα/β (top), total IKKβ (middle), and β-tubulin loading control (bottom). (B) Densitometric quantification of phosphorylated IKKα/β relative to total IKKβ and β-tubulin. (C) Representative immunoblots of HUVEC treated as under panel A, but stimulated with IL-1β for 15 or 30 minutes. Panels show phosphorylated JNK (top), total JNK (middle), and β-tubulin loading control (bottom). (D) Densitometric quantification of phosphorylated JNK relative to total JNK and β-tubulin. (E) Representative immunoblots of HUVEC stimulated with TNF (1 ng/ml) for 0, 5, and 60 minutes in the presence (+) and absence (-) of 3PO (20 μM) 30 minutes pretreatment. Panels show phosphorylated IKKα/β (top), total IKKβ (middle), and β-tubulin loading control (bottom). (F) Densitometric quantification of phosphorylated IKKα/β relative to total IKKβ and β-tubulin. (G) Representative immunoblots of HUVEC stimulated with TNF (1 ng/ml) for 0, 15, and 30 minutes in the presence (+) and absence (-) of 3PO (20 μM) pretreatment for 30 minutes. Panels show phosphorylated JNK (top), total JNK (middle), and β-tubulin loading control (bottom). (H) Densitometric quantification of phosphorylated JNK relative to total JNK and β-tubulin. Densitometry plots show means +/-SD of four (D, F, H) or five (B) independent experiments, ** p<0.01, **** p<0.0001, two-tailed Student’s t-test. Full-length blots are shown in S1 Fig.

We previously showed that 3PO inhibits the IL-1β-induced phosphorylation of p65 and IκBα, and thereby signaling through the NFκB pathway, in endothelial cells [7]. To understand if the inhibition was mediated at the level of the individual phosphorylation events or by inhibition of upstream signaling, we asked if 3PO also modulated IL-1β-induced phosphorylation of the upstream kinases IKKα and IKKβ (IKKα/β) at Serine 176-177/180-181. Such phosphorylation results in activation of the IKK complex, which is a key event in activation of the NFκB pathway [20, 21]. HUVECs were treated with 3PO or vehicle for 30 minutes before stimulation with recombinant human IL-1β (1 ng/ml) for 5 and 60 minutes, respectively. Phosphorylation levels of IKKα/β were determined by immunoblotting followed by quantification by densitometric scanning and normalization to total IKKβ and β-tubulin. We observed (Fig 1A and 1B) that 3PO treatment reduced phosphorylation of IKKα/β 5 minutes after IL-1β stimulation. After 60 minutes, however, cells appeared less sensitive to 3PO treatment, indicating that the effect of 3PO was transient.

Stimulation of the IL-1 receptor activates the IKK complex via MyD88- and IRAK-dependent formation of an active TRAF6/TAK1/TAB2 complex [22–24] that also mediates activation of c-jun-N-terminal kinases (JNK) [24]. To determine if 3PO, in addition to its effect on IKK/NFκB, modulated IL-1β-signaling through the stress-activated kinase pathway, we next monitored IL-1β-induced phosphorylation of c-jun N-terminal kinase (JNK) at threonine 183 and tyrosine 185 [25, 26]. Again, HUVECs were treated with 3PO (20 μM) for 30 minutes and stimulated with IL-1β for 15 and 30 minutes, and phosphorylation of JNK was determined by immunoblotting and densitometric quantification and normalization to total JNK and β-tubulin. We observed (Fig 1C and 1D) that 3PO significantly reduced the IL-1β-induced phosphorylation of JNK both 15 and 30 minutes after stimulation. Together, these results showed that 3PO inhibited phosphorylation of two pivotal signaling pathways activated by IL-1β in endothelial cells.

Similar to IL-1β, stimulation with tumor necrosis factor (TNF) elicits NFκB and JNK activity in endothelial cells [27]. However, in contrast to IL-1β, TNF-induced activation of the TAK1/TAB2 complex depends on RIP1, TRADD and TRAF2/5 [28]. We next tested if 3PO also influenced TNF-induced phosphorylation of IKKα/β and JNK. HUVECs were treated with 3PO (20 μM) or vehicle for 30 minutes and stimulated with TNF for 5, 30, and 60 minutes, respectively. Phosphorylation of IKKα/β was determined at 5 and 60 minutes (Fig 1E and 1F) and phosphorylation of JNK at 15 and 30 minutes post-TNF stimulation (Fig 1G and 1H). We observed that 3PO reduced phosphorylation of IKKα/β after 5, but not 60, minutes of TNF stimulation (Fig 1E and 1F). Similarly, TNF-dependent JNK phosphorylation was reduced after 15, but not 30, minutes of TNF stimulation (Fig 1G and 1H). Our findings show that 3PO inhibits inflammatory signaling induced by both IL-1β and TNF in a similar manner.

Because high doses of 3PO have potential to cause toxicity in endothelial cells [17], we also evaluated cell death by measuring leakage of endogenous lactate dehydrogenase (LDH) in the cell culture medium and observed no evidence of membrane disruption (S2 Fig).

### Ablation of PFKFB3 does not reduce IL-1β induced expression of vascular adhesion molecules

**Fig 2:**
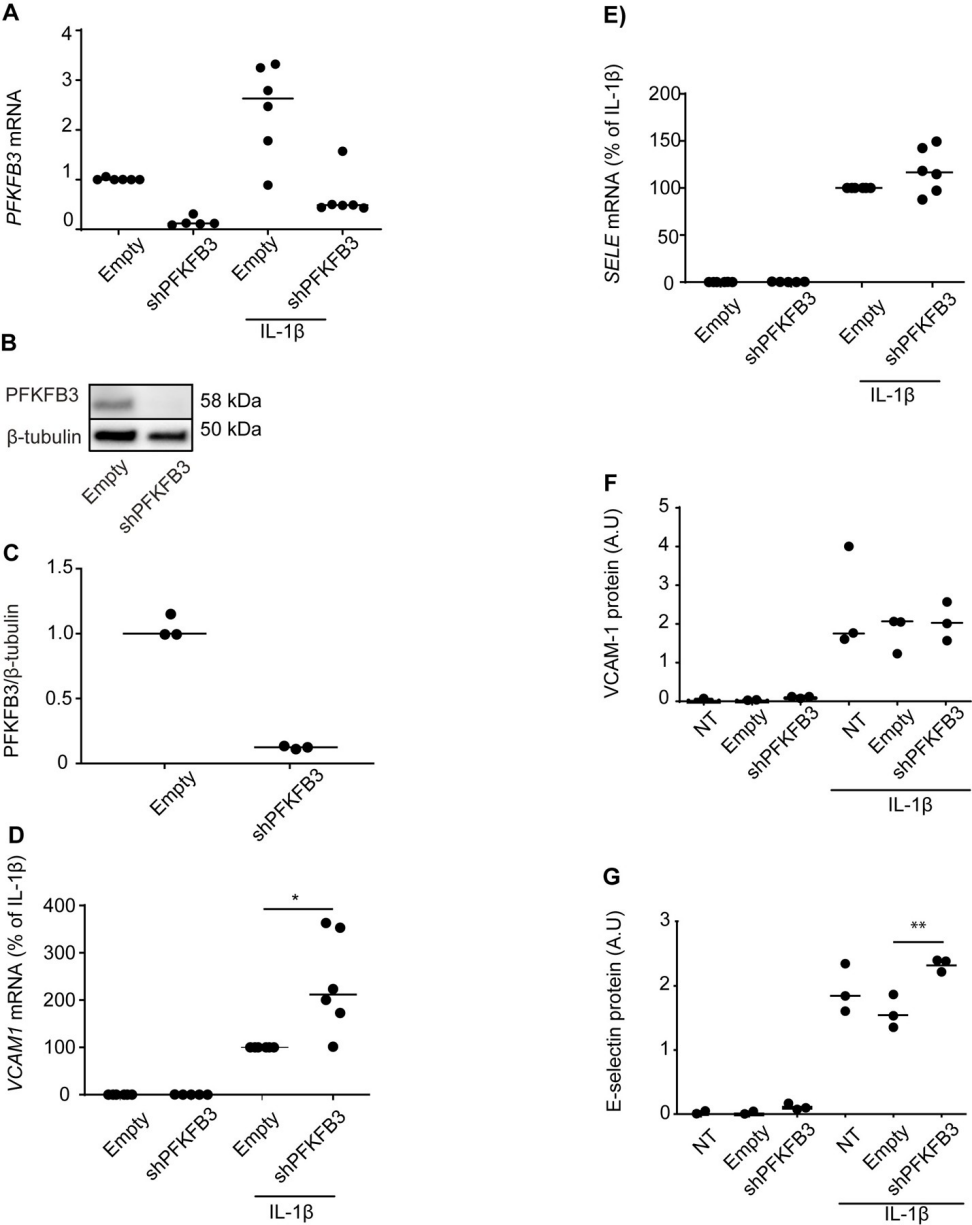
PFKFB3-silencing does not inhibit IL-1β-induced upregulation of vascular adhesion molecules. HUVECs were transduced with empty vector (Empty) or vectors expressing shRNA targeting PFKFB3 (shPFKFB3) as described in material and methods and analyzed for PFKFB3-expression (A-C), or stimulated with IL-1β (1 ng/ml, 3 hours) and analyzed for expression of VCAM-1 (D, F) and SELE (E, G) by RT-qPCR (C-D) and cell ELISA (E-F). In A, C, and D, relative expression is calculated by 2^-ΔΔCT, using *HPRT* as a reference gene and medium-treated (A) or IL-1β-stimulated (C and D) cells transduced with empty vectors as controls. Means of 3–6 independent experiments are shown as individual data points, with bars showing the net mean, * p<0.05, two-tailed Student’s t-test (cell ELISA) or Wilcoxon signed ranked test (RT-qPCR). Full-length blot of (B) is shown in S4 Fig.

To substantiate the effects of PFKFB3-inhibition on inflammatory endothelial activation, we next silenced the enzyme by lentiviral transduction with shRNA specific to *PFKFB3*. Knockdown efficiency was determined by RT-qPCR (Fig 2A) and immunoblotting (Fig 2B and 2C) and achieved silencing levels that have previously been shown to inhibit proliferation and angiogenic sprouting [2] comparable to 3PO treatment [10]. We then stimulated *PFKFB3*-silenced HUVECs with IL-1β (1 ng/ml, 3 hours) and measured the expression of mRNA encoding VCAM-1 and E-selectin by RT-qPCR. In contrast to 3PO treatment (20 μM) [7], *PFKFB3* RNA interference inhibited neither *VCAM1* (Fig 2D) nor *SELE* (Fig 2E) expression. In fact, *VCAM1* mRNA was upregulated by silencing PFKFB3 (Fig 2D). Findings were confirmed at the protein level using a cell ELISA where levels of immunoreactive proteins in fixed, adherent endothelial cells were quantified and normalized to cell number using crystal violet [19] (Fig 2F and 2G). This was further confirmed by immunoblot for phosphorylation of IKKα/β (S3A Fig), JNK (S3B Fig), and VCAM-1 (S3C Fig). We further confirmed by cell ELISA that E-selectin and VCAM-1 expression was not significantly affected by PFKFB3-silencing 3, 6, or 24 hours post IL-1β stimulation (S3D and S3E Fig) or 3, 6, or 24 hours post stimulation with TNF (1 ng/ml) (S3F and S3G Fig). In conclusion, inhibition of PFKFB3 expression does not mirror the effect of 3PO on adhesion molecule expression.

### Inhibition of PFKFB3 by YN1 does not reduce IL-1β-induced NFκB activation

**Fig 3:**
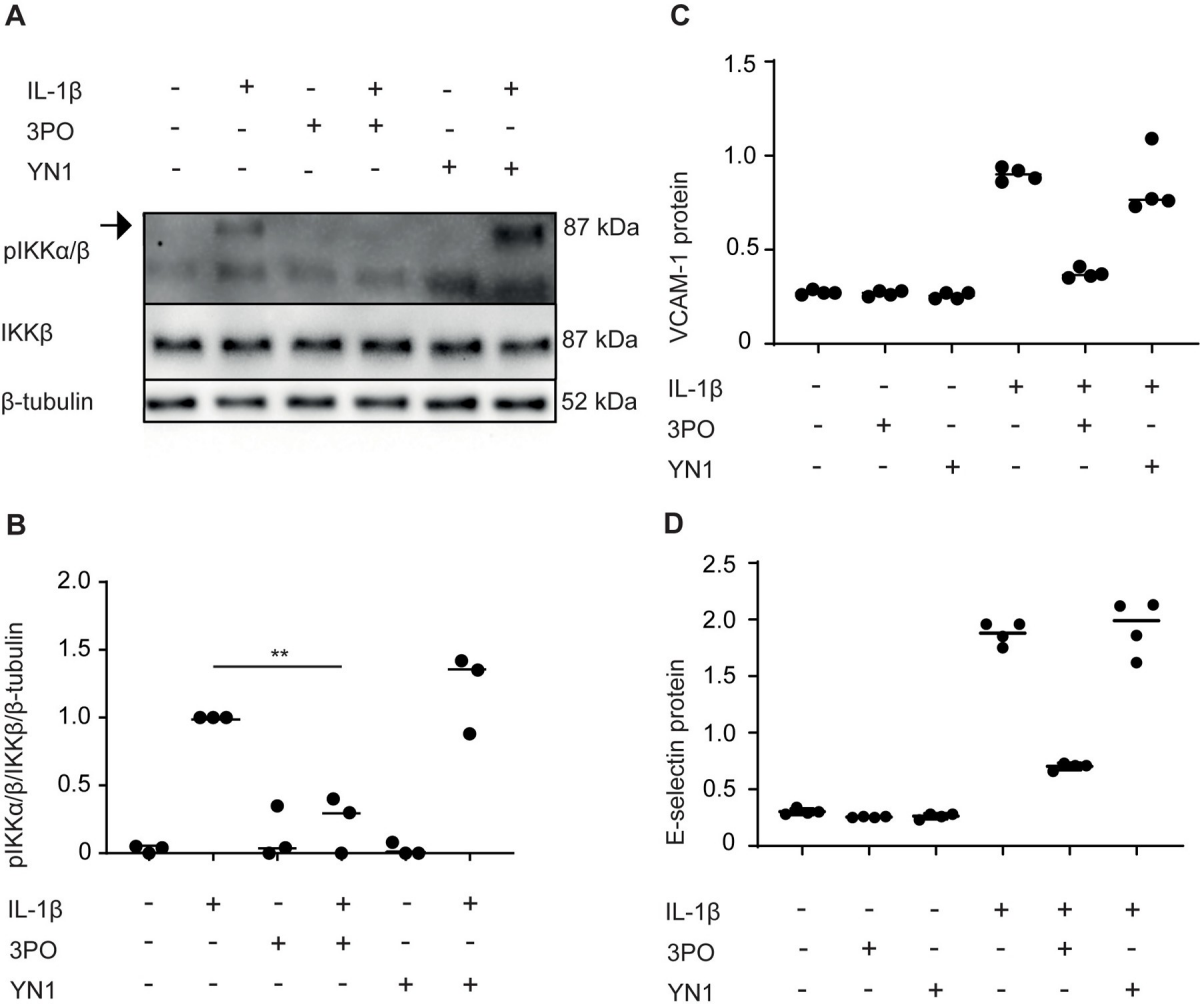
YN1 does not reduce inflammatory activation of endothelial cells. (A) Representative immunoblots of cell extracts from HUVECs stimulated with IL-1β (1 ng/ml) for 0 or 5 minutes after pretreatment for 30 minutes with medium, 3PO (20 μM), or YN1 (20 μM). Panels show phosphorylated IKKα/β (top), total IKKβ (middle), and β-tubulin loading control (bottom). Full-length version of another blot in the experimental series is shown in S6 Fig. (B) Densitometric quantification of phosphorylated IKKα/β relative to total IKKβ and β-tubulin. The graph show means of three independent experiments, ** p<0.01, Student’s t-test. (C and D), HUVEC were stimulated with IL-1β (1 ng/ml) for 3 hours in the absence or presence of 3PO (20 μM) or YN1 (20 μM). Relative protein levels of VCAM-1 (C) and E-selectin (D) were determined by cell ELISA. Graphs are representative of three independent experiments.

We next evaluated if the effects of 3PO could be reproduced by YN1, an alternative pharmacological inhibitor that inhibits glycolysis and angiogenic behavior in endothelial cells in a manner similar to 3PO [7, 9, 10]. In contrast to 3PO, YN1 did not reduce IKKα/β-phosphorylation in response to IL-1β (Fig 3A and 3B). Cells were also treated with YN1 (20 μM) before stimulation with TNF (1 ng/ml) for 5 or 60 minutes and for 15 and 30 minutes (S4B Fig). Again, YN1 did not reduce TNF-induced phosphorylation of IKKα/β or JNK (S5A and S5B Fig). To further substantiate these observations, HUVECs were treated with 3PO or YN1 and stimulated with IL-1β (1 ng/ml, 3 hours), and the upregulation of VCAM-1 and E-selectin was assessed by cell ELISA (Fig 3C and 3D). We found that YN1 reduced neither VCAM-1 nor E-selectin, and this was confirmed by immunoblot at 3 and 6 hours (S4C Fig). 3PO in contrast inhibited both VCAM-1 and E-selectin expression (Fig 3C and 3D). In summary, the alternative PFKFB3 inhibitor YN1 does not reproduce the inhibitory effect of 3PO on inflammatory endothelial activation, strengthening the assumption that this effect of 3PO is not mediated by PFKFB3 inhibition.

### 3PO sustains its effects on adhesion molecule expression after knockdown of PFKFB3

**Fig 4:**
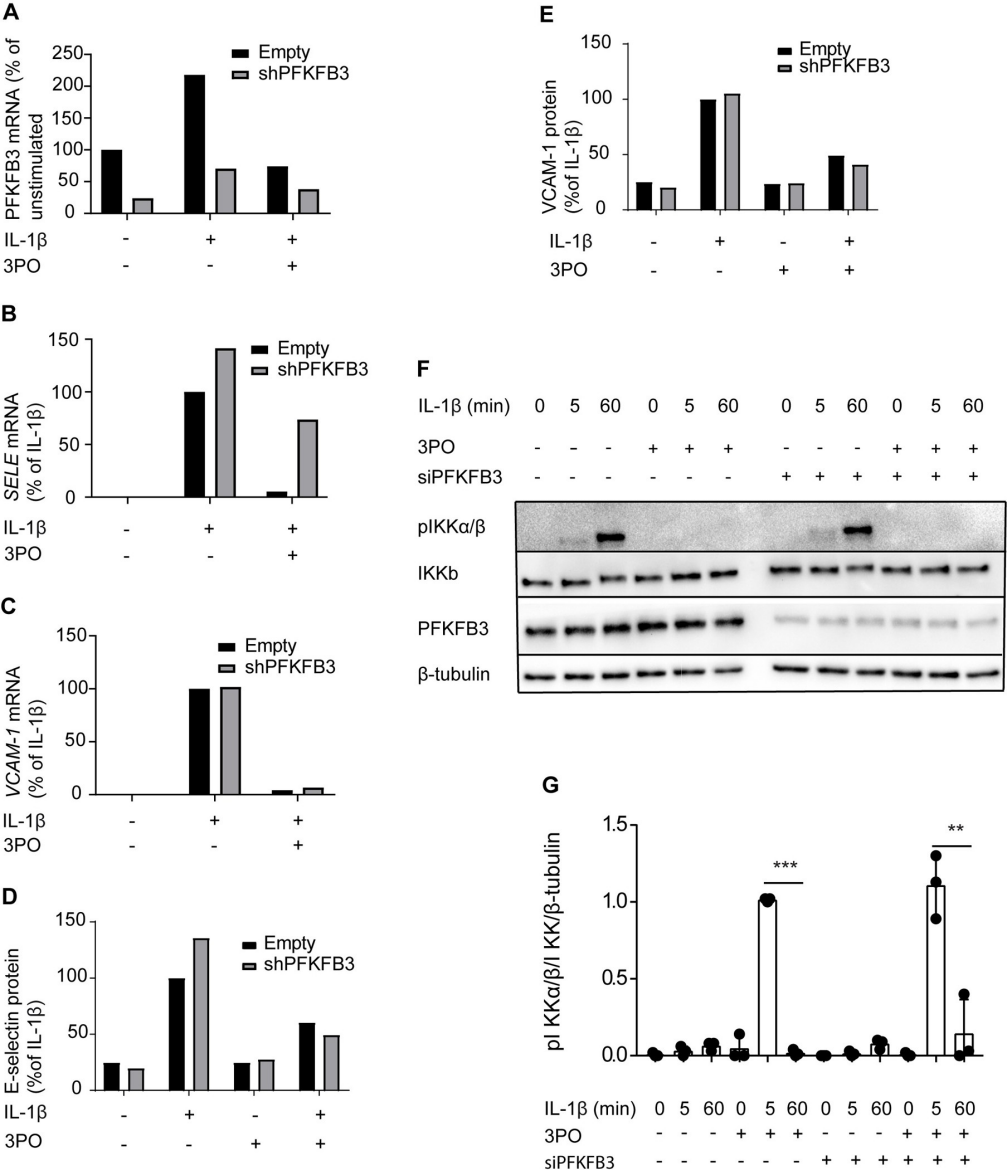
3PO sustains its effects on adhesion molecule expression after knockdown of PFKFB3. HUVEC were transduced with control lentivirus (Empty) or lentivirus containing shRNA targeting PFKFB3 (shPFKFB3) then stimulated with IL-1β (1 ng/ml) for 3 hours in the absence or presence of 3PO (20 μM. Relative mRNA levels of PFKFB3 (A), E-selectin (B) and VCAM-1 was determined by RT-qPCR. Relative protein levels of E-selectin (D) and VCAM-1 (E) were determined by cell ELISA. Graphs are representative of two independent experiments. (F) Representative immunoblots of cell extracts from HUVECs transfected with non-targeting siRNA (Scramble) or siRNA targeting PFKFB3 (siPFKFB3) stimulated with IL-1β (1 ng/ml) for 0, 5 or 60 minutes after pretreatment for 30 minutes with medium or 3PO (20 μM). Panels show phosphorylated IKKα/β (top), total IKKβ (second from the top), PFKFB3 (third from the top), and β-tubulin loading control (bottom). (G) Densitometric quantification of phosphorylated IKKα/β relative to total IKKβ and β-tubulin. The graph show means of three independent experiments, ** p<0.01, *** p<0.001, Student’s t-test.

We next evaluated the effect of 3PO on IL-1β-stimulated activation of PFKFB3-silenced cells. PFKFB3-silenced cells were treated with 3PO (20 μM), then stimulated with IL-1β (1 ng/ml) for 3 hours, and measured mRNA (Fig 4A and 4B) and protein (Fig 4C and 4D) expression of E-selectin (Fig 4A and 4C) and VCAM-1 (Fig 4B and 4D). Strengthening the evidence for PFKFB3-independent effects of 3PO, we found that knockdown of PFKFB3 did not obliterate the effect of 3PO on upregulation of adhesion molecules. Moreover, the effect of 3PO on IL-1β-induced phosphorylation of IKKa/β persisted in PFKFB3-silenced cells (Fig 4E and 4F). Together, these observations support the notion that the effects of 3PO on inflammatory activation may arise through other mechanisms than PFKFB3-inhibition.

### 3PO and YN1 has no detectable effects on HUVEC ECAR and OCR in the relevant time window of our experimental setup

**Fig 5:**
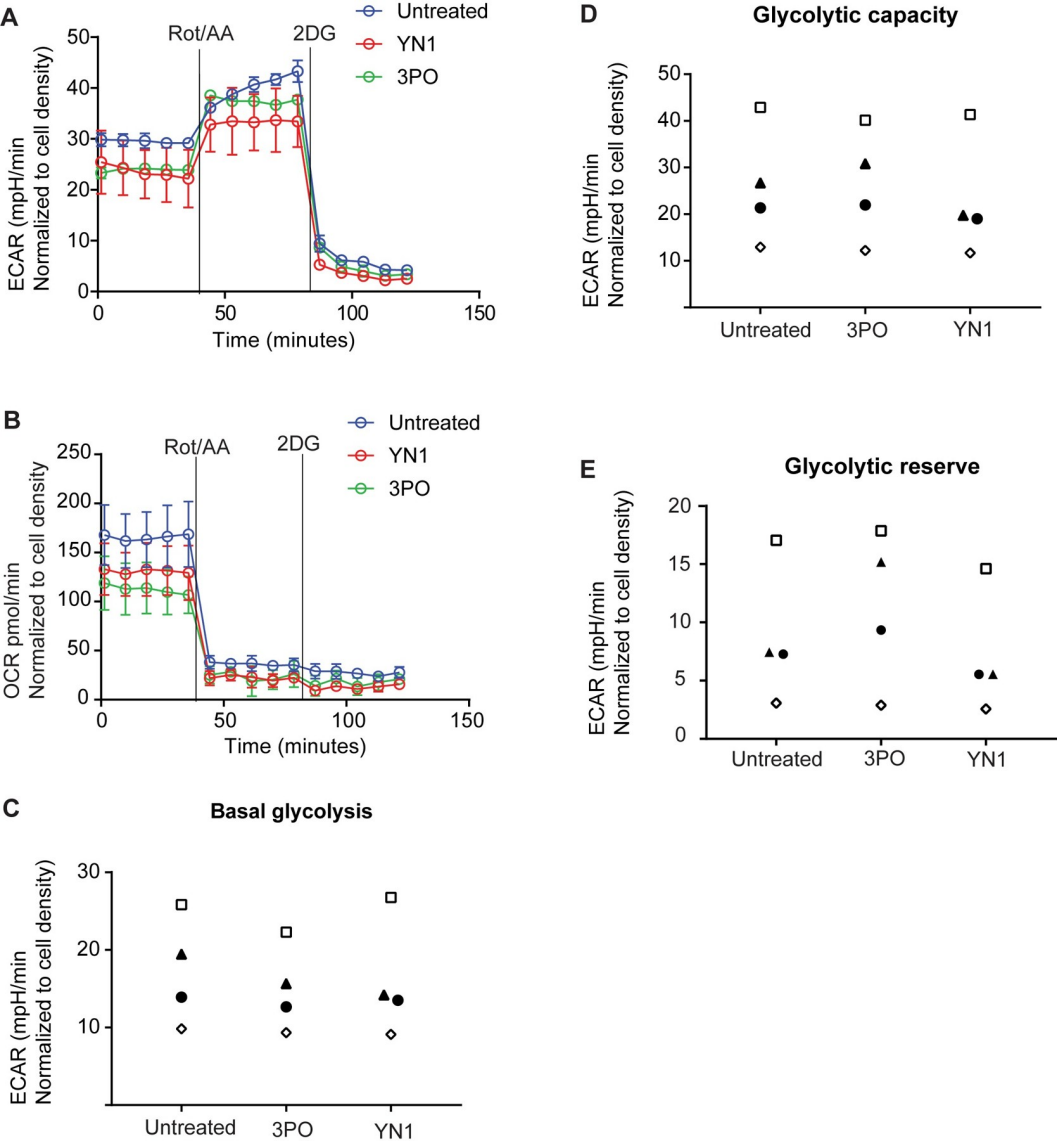
Short term administration of 3PO or YN1 has no marked effect on ECAR and OCR in HUVECs. Extracellular flux analysis using the Seahorse XFe 24 system to measure ECAR (A) and OCR (B) one hour after treatment with 3PO (20 μM) or YN1 (20 μM). Values for basal glycolysis (C), glycolytic capacity (D), and glycolytic reserve (E) are shown for four independent experiments. Individual experiments are represented by the same symbol in all three panels.

Previous experiments demonstrating the effect of 3PO and YN1 on glycolysis in HUVECs measured glycolytic rates after 6 hours incubation with the inhibitors [10]. In our experimental setup, cells were stimulated with inflammatory cytokines as soon as 30 minutes after administration of the small inhibitors. To ensure that the difference between 3PO and YN1 was not simply due to a more rapid onset of 3PO activity, we evaluated the effect of 3PO and YN1 on glycolytic flux and mitochondrial activity one hour after administration, which is a relevant time window for our experimental setup. ECAR and OCR rates were measured by the Seahorse glycolytic rate assay (Agilent), showing only minimal effects of 3PO and YN1 on measures of cellular metabolism at this early time point (Fig 4A and 4B). Moreover, repeated experiments showed no consistent reduction in basal glycolysis or glycolytic capacity (Fig 5C and 5D). To this end, 3PO and YN1 did not influence the glycolytic reserve (Fig 5E) 1 hour after administration. We also explored the short-term effects of IL-1β on glycolysis by injecting IL-1β into the wells using the Seahorse system. This showed that IL-1β did not significantly affect ECAR (S7A and S7B Fig) nor OCR (S7C Fig) in the time window studied. Taken together, this lack of immediate effect on glycolysis supports the assumption that the effects of 3PO on downstream events of IL-1β are not mediated by inhibition of PFKFB3 and cellular glycolysis.

## Discussion

We here dissected the inhibition of inflammatory endothelial cell activation by 3PO and revealed that it is unlikely to be directly linked to inhibition of PFKFB3 activity or glycolysis.

We found that 3PO inhibited the activation of both NFκB and stress-kinase activated signaling cascades in human endothelial cells, thus dampening IL-1β-induced endothelial activation. 3PO also modulated inflammatory activation in response to TNF, another prototypic inflammatory cytokine. IL-1β and TNF both induce activation of the common TAK1/TAB2 complex upon association with their respective receptors, but via different upstream paths. While IL-1 receptor activation results in signaling through MyD88, IRAK, and TRAF6 upstream of TAK1/TAB2, TNF signaling depends on RIP1, TRADD, and TRAF2/5 [22, 23, 28]. Downstream to TAK1/TAB2 complex activation, the NFκB and stress-kinase activated pathways diverge upon phosphorylation of IKKα/β and JNK, respectively. However, both pathways contribute to the transcription of inflammatory genes, in endothelial cells including adhesion molecules with important roles in leukocyte recruitment.

The simultaneous inhibition of JNK and IKKα/β activation by 3PO is therefore unlikely to result from direct interference with a single signaling event, unless this occurs at the level of TAK1/TAB2. Alternatively, our observations could be explained by 3PO interfering with signaling in a more general manner, for example by modulating cellular processes like membrane/cytoskeletal dynamics or phosphorylation, or by affecting the activity of enzymes that exert cross-talk with inflammatory signaling at several levels.

However, at this point of our investigation, we assessed if the effects of 3PO on inflammatory endothelial activation could be reproduced by silencing its intended target, *PFKFB3*. We found that silencing *PFKFB3* caused no reduction in cytokine-stimulated upregulation of adhesion molecules and therefore did not mirror the anti-inflammatory effect of 3PO. In fact, *VCAM1* transcription increased slightly in *PFKFB3*-silenced cells. The reason for this increase remains unknown. However, the effect was not mirrored by a change in protein expression and is unlikely to be of functional relevance. Nevertheless, the inconsistency between 3PO and genetic inhibition of PFKFB3 made us question whether the actions of 3PO in our system reflected other mechanisms than modulation of PFKFB3 activity.

Knockdown using shRNA and siRNA is often associated with incomplete ablation of protein, and a valid consideration is therefore whether the activity of residual PFKFB3 could have obscured our results. Notably, the reduction of PFKFB3 expression in our experiments was comparable to levels previously found to mimic the inhibition of proliferation and sprouting exerted by 3PO in HUVEC [2, 7, 10]. Nevertheless, PFKFB3 was still induced to some extent by inflammatory stimulation (as shown in Fig 2A), and to completely exclude the possibility of residual PFKFB3 activity, experiments in endothelial cells from *Pfkfb3*-deficient mice would be required. On the other hand, the use of *Pfkfb3*-deficient mice would fail to address another possible cause of the discrepancy, which is the different kinetics of PFKFB3 inhibition exerted by pharmacological and genetic approaches. While pharmacological inhibition by 3PO only transiently reduces glycolysis [10], genetic silencing permanently impairs PFKFB3 function and is therefore likely to differ from 3PO in its effects on metabolic intermediates and metabolic fluxes. For example, acute application of 3PO to a cell with active glycolysis can be assumed to result in accumulation of fructose-6-phosphate and increased flux through the hexosamine biosynthesis pathway. Indeed, one could speculate that such a redirection of metabolites might have potential to influence phosphorylation events through changes in O-GlcNacylation, either directly [29] or by activation of O-GlcNacylation-activated kinases like CaMKII kinase [30].

To circumvent this difference in kinetics and more faithfully reproduce the expected 3PO-mediated inhibition of PFKFB3, we targeted PFKFB3 by an alternative small molecule inhibitor, YN1. Similar to 3PO, YN1 inhibits PFKFB3-dependent formation of fructose 2,6-Bis phosphate [2, 9, 10, 31]. Nevertheless, pretreatment with YN1 reduced neither the IL-1β-induced activation of the NFκB pathway nor the upregulation of VCAM-1 and E-selectin in human endothelial cells. Notably, 3PO sustained its inhibitory effect on the activation of the NFκB pathway and upregulation of adhesion molecules even when PFKFB3 expression was knocked down.

Neither 3PO nor YN1 significantly influenced basal ECAR or OCR within the time frame used in our experimental setup. We suggest that the effects of 3PO and Y1 would have been better appreciated through measuring endogenous levels of fructose2,6,bisphophate, despite this being an indirect way of measuring PFKFB3 activity. We also found that IL-1β did not significantly affect metabolism in the timeframe relevant to the phosphorylation of IKKα/β or JNK. Other studies have also demonstrated that pharmacological PFKFB3 inhibition has minimal effect on ECAR [31]. Moreover, the maximal inhibition of glycolysis by 3PO reported by extracellular tracking of H^3^-labelled glucose (35–40%, 6 hours post administration [10] relied on a high dose of 3PO (40 μM) that also bears significant potential to disintegrate endothelial junctions [17]. Most cell culture experiments, including ours, therefore employ a lower dose of 3PO (20 μM) that inhibited glycolytic rates to a much lesser extent [10]. Nevertheless, it is possible that a more pronounced effect on ECAR could have been achieved also at early time points by carefully titrating the dose of 3PO and cell culture conditions.

However, and despite effects on extracellular acidification being at best minimal in our experimental setup, 3PO consistently inhibited inflammatory activation in the same conditions. This suggests that the anti-inflammatory effect of 3PO in endothelial cells is unlikely to result from a reduction in pan-cellular glycolysis and/or rerouting of metabolites alone, again supporting that this effect is independent of the activity of PFKFB3.

3PO reduces disease severity in several mouse models of inflammation, including colitis, psoriasis-like dermatitis, and sepsis [10, 11]. To our knowledge, these studies have not been extended to include the use of alternative PFKFB3-inhibitors or genetic modulation of Pfkfb3. Nevertheless, our findings do not preclude that the anti-inflammatory effect of 3PO in murine inflammation could be mediated by Pfkfb3-inhibition, either by its effect on pathological angiogenesis [10] or by inhibiting the activation of other cell types relying on glycolysis for efficient effector function. Similarly, to determine whether the anti-inflammatory effect of 3PO in activated synovial fibroblasts [13, 14] is off-target as in endothelial cells or mediated by PFKFB3 inhibition, requires further experimentation. Emphasizing this point, hypoxic macrophages rely on PFKFB3 expression for their inflammatory activation [12]. Moreover, a recent publication reported that silencing of PFKFB3 reduces TNF-induced inflammatory activation of EA.hy926 cells [32]. While our study does not pinpoint the reason for the discrepancy between our results and the reported findings in this endothelial-like cell line, it is important to realize that EA.hy926 was originally generated by fusing HUVECs with the A549 human lung carcinoma cell line [33] and is not necessarily representative of patient-derived primary endothelial cells. The difference between EA.hy296 and HUVEC can be exemplified by the tolerance to 3PO, as optimal inhibition of inflammatory activation in EA.hy296 required concentrations of 3PO that would be toxic to primary HUVECs [17, 32].

In conclusion, we show that 3PO inhibits early events of inflammatory endothelial cell activation in response to two prototypic inflammatory cytokines. The anti-inflammatory effect could not be reproduced by genetic silencing or alternative PFKFB3-inhibition. Moreover, 3PO inhibited inflammation even in the absence of obvious changes in ECAR. Our findings therefore indicate that manipulation of PFKFB3 activity in primary human endothelial cells does not inhibit inflammatory activation in itself, and suggest that 3PO exerts its effects by other means.

## Supporting information

S1 Fig Full-length immunoblots of membranes from Fig 1. (A) Representative full-length immunoblots of HUVEC stimulated with IL-1β (1 ng/ml) for 0, 5, and 60 minutes in the presence (+) or absence of 3PO (20 μM) pretreatment for 30 minutes. Panels show phosphorylated IKKα/β (top) (mAb clone 16A6), total IKKβ (mAb clone D30C6) (middle), and β-tubulin loading control (rabbit polyclonal) (bottom). Two bands are visible on the membrane, with the lower band being unaffected by stimulation with IL-1β and most likely unspecific. After imaging of pIKKα/β, the membrane was stripped as detailed in materials and methods and cut between the 52 and 76 kDa marker before staining for total IKKβ and β-tubulin. (B) Representative full-length immunoblots of HUVECs treated as in panel A. Panels show phosphorylated JNK (top) (rabbit polyclonal), total JNK (middle) (rabbit polyclonal), and β-tubulin loading control (bottom) (rabbit polyclonal). The same membrane was used to stain for phosphorylated JNK, total JNK, and β-tubulin, in that order with stripping between staining. Two bands correspond to the estimated sizes of JNK (46 and 56 kDa) in addition to unspecific bands of higher molecular weight. (C) Representative full-length immunoblots of HUVEC stimulated with TNF (1 ng/ml) for 0, 5, and 60 minutes in the presence (+) and absence (-) of 3PO (20 μM) 30 minutes pretreatment. Panels show phosphorylated IKKα/β (top), total IKKα/β (middle), and β-tubulin loading control (bottom). Membranes were cut between the 52 and 76 kDa marker after imaging pIKKα/β. (D) Representative full-length immunoblots of HUVEC stimulated with TNF (1 ng/ml) for 0, 15, and 30 minutes in the presence (+) and absence (-) of 3PO (20 μM) pretreatment for 30 minutes Panels show phosphorylated JNK (top), total JNK (middle), and β-tubulin loading control (bottom).
(TIF)

S2 Fig 3PO treatment does not cause notable loss of membrane integrity and leakage of lactate dehydrogenase (LDH). HUVECs were stimulated with IL-1β (1 ng/ml) in the presence or absence of 3PO (20 μM) for 1 or 6 hours. Supernatants were collected and toxicity was measured using the Cytotoxicity Detection Kit^PLUS^ (LDH) (Sigma 4744926001) according to the manufacturer’s instructions. LDH levels were normalized to control (n = 2).
(TIF)

S3 Fig Ablation of PFKFB3 does not reduce activation of NFκB or JNK pathways or expression of adhesion molecules. Representative blots (n = 2) of HUVECs transduced with lentivirus without shRNA (Empty) or lentivirus expressing shRNA against PFKFB3 (shPFKFB3) then stimulated with IL-1β for 5 and 60 minutes for phosphorylation IKKα/β (A), 15 or 30 minutes for phosphorylation of JNK (B) or 3 hours for protein level of VCAM-1 (C). A: Panels show phosphorylated IKKα/β (top) (mAb clone 16A6), total IKKβ (mAb clone D30C6) (middle), and β-tubulin loading control (rabbit polyclonal) (bottom) B: Panels show phosphorylated JNK (top) (rabbit polyclonal), total JNK (middle) (rabbit polyclonal), and β-tubulin loading control (bottom) (rabbit polyclonal). C: Panels show VCAM-1 (top) (polyclonal goat) β-tubulin (bottom) (polyclonal rabbit). Representative cell ELISA experiment (n = 2) showing the IL-1β-induced (1 ng/ml) upregulation of E-selectin (D) or VCAM-1 (E) or TNF-induced (1 ng/ml) upregulation of E-selectin (F) or VCAM-1 (G) after 3, 6 and 24 hours of stimulation in control cells (Empty) and in PFKFB3 knockdown cells (shPFKFB3).
(TIF)

S4 Fig Full-length immunoblot of membrane from Fig 2. Representative full-length immunoblot of HUVECs transduced with lentivirus without shRNA (Empty) or lentivirus expressing shRNA against PFKFB3 (mAb clone EPR12594) (shPFKFB3). β-tubulin (rabbit polyclonal) was used as loading control. The membrane was stripped after imaging PFKFB3 and stained for β-tubulin.
(TIF)

S5 Fig Inhibition of PFKFB3 by YN1 does not reduce activation of NFκB or JNK pathways nor upregulation of VCAM-1. A: Representative immunoblots of cell extracts from HUVECs stimulated with IL-1β (1 ng/ml) for 0, 5 or 60 minutes after pretreatment for 30 minutes with medium or YN1 (20 μM). Panels show phosphorylated IKKα/β (top), total IKKβ (middle), and β-tubulin loading control (bottom). B: Representative immunoblot (n = 2) of cell extracts from HUVECs stimulated with IL-1β (1 ng/ml) for 0, 15 or 30 minutes after pretreatment for 30 minutes with medium or YN1 (20 μM). Panels show phosphorylated JNK (top) (rabbit polyclonal), total JNK (middle) (rabbit polyclonal), and β-tubulin loading control (bottom) (rabbit polyclonal). C: Representative immunoblot (n = 2) of cell extracts from HUVECs stimulated with IL-1β (1 ng/ml) in the presence or absence of 3PO (20 μM) or YN1 (20 μM) for 3 or 6 hours. Panels show VCAM-1 (top) (polyclonal goat) β-tubulin (bottom) (rabbit polyclonal).
(TIF)

S6 Fig Full-length immunoblots of membrane from Fig 3. Representative full-length immunoblots of cell extracts from HUVECs stimulated with IL-1β (1 ng/ml) for 0 (-) or 5 (+) minutes without (-) or with (+) pretreatment with either 3PO (20 μM), or YN1 (20 μM) for 30 minutes. Panels show phosphorylated IKKα/β (top) (mAb clone 16A6), total IKKβ (mAb clone D30C6) (middle), and β-tubulin loading control (rabbit polyclonal) loading control (bottom). There is a band of slightly smaller molecular weight than expected visible in all lanes for pIKKα/β that seems to be unaffected by either IL-1β stimulation or treatment with 3PO or YN1 and is likely unspecific. The same membrane was used to stain for phosphorylated IKKα/β, total IKKβ, and β-tubulin, in that order with stripping between staining.
(TIF)

S7 Fig Short-term stimulation with IL-1β does not significantly affect ECAR in HUVECs. Representative (n = 2) seahorse experiment showing untreated HUVECs (untreated) or treated with 3PO (20 μM) or YN1 (20 μM) for 1 hour. IL-1β or DMEM (med) is injected at 1, rotenone/antimycin A at 2 and 2-deoxyglucose at 3 then ECAR (A) and ECAR was compared in in cells stimulated with IL-1β compared to non-stimulated cells (B) and OCR was meassured. Representative of 2 independent experiments.
(TIF)

## References

[1] O'Neill LA, Kishton RJ, Rathmell J. A guide to immunometabolism for immunologists. Nat Rev Immunol. 2016;16(9):553–65. 10.1038/nri.2016.70 27396447 PMC5001910

[2] De Bock K, Georgiadou M, Schoors S, Kuchnio A, Wong BW, Cantelmo AR, et al Role of PFKFB3-driven glycolysis in vessel sprouting. Cell. 2013;154(3):651–63. 10.1016/j.cell.2013.06.037 23911327

[3] Van Schaftingen E, Lederer B, Bartrons R, Hers HG. A kinetic study of pyrophosphate: fructose-6-phosphate phosphotransferase from potato tubers. Application to a microassay of fructose 2,6-bisphosphate. European journal of biochemistry. 1982;129(1):191–5. 10.1111/j.1432-1033.1982.tb07039.x 6297885

[4] Yalcin A, Clem BF, Imbert-Fernandez Y, Ozcan SC, Peker S, O'Neal J, et al 6-Phosphofructo-2-kinase (PFKFB3) promotes cell cycle progression and suppresses apoptosis via Cdk1-mediated phosphorylation of p27. Cell Death Dis. 2014;5:e1337 10.1038/cddis.2014.292 25032860 PMC4123086

[5] Clem BF, O'Neal J, Tapolsky G, Clem AL, Imbert-Fernandez Y, Kerr DA 2nd, et al Targeting 6-phosphofructo-2-kinase (PFKFB3) as a therapeutic strategy against cancer. Mol Cancer Ther. 2013;12(8):1461–70. 10.1158/1535-7163.MCT-13-0097 23674815 PMC3742633

[6] Xu Y, An X, Guo X, Habtetsion TG, Wang Y, Xu X, et al Endothelial PFKFB3 plays a critical role in angiogenesis. Arterioscler Thromb Vasc Biol. 2014;34(6):1231–9. 10.1161/ATVBAHA.113.303041 24700124 PMC4120754

[7] Cantelmo AR, Conradi LC, Brajic A, Goveia J, Kalucka J, Pircher A, et al Inhibition of the Glycolytic Activator PFKFB3 in Endothelium Induces Tumor Vessel Normalization, Impairs Metastasis, and Improves Chemotherapy. Cancer Cell. 2016;30(6):968–85. 10.1016/j.ccell.2016.10.006 27866851 PMC5675554

[8] Clem B, Telang S, Clem A, Yalcin A, Meier J, Simmons A, et al Small-molecule inhibition of 6-phosphofructo-2-kinase activity suppresses glycolytic flux and tumor growth. Mol Cancer Ther. 2008;7(1):110–20. 10.1158/1535-7163.MCT-07-0482 18202014

[9] Seo M, Kim JD, Neau D, Sehgal I, Lee YH. Structure-based development of small molecule PFKFB3 inhibitors: a framework for potential cancer therapeutic agents targeting the Warburg effect. PLoS One. 2011;6(9):e24179 10.1371/journal.pone.0024179 21957443 PMC3177832

[10] Schoors S, De Bock K, Cantelmo AR, Georgiadou M, Ghesquiere B, Cauwenberghs S, et al Partial and transient reduction of glycolysis by PFKFB3 blockade reduces pathological angiogenesis. Cell metabolism. 2014;19(1):37–48. 10.1016/j.cmet.2013.11.008 24332967

[11] Gong Y, Lan H, Yu Z, Wang M, Wang S, Chen Y, et al Blockage of glycolysis by targeting PFKFB3 alleviates sepsis-related acute lung injury via suppressing inflammation and apoptosis of alveolar epithelial cells. Biochem Biophys Res Commun. 2017;491(2):522–9. 10.1016/j.bbrc.2017.05.173 28576491

[12] Tawakol A, Singh P, Mojena M, Pimentel-Santillana M, Emami H, MacNabb M, et al HIF-1alpha and PFKFB3 Mediate a Tight Relationship Between Proinflammatory Activation and Anerobic Metabolism in Atherosclerotic Macrophages. Arterioscler Thromb Vasc Biol. 2015;35(6):1463–71. 10.1161/ATVBAHA.115.305551 25882065 PMC4441599

[13] Biniecka M, Canavan M, McGarry T, Gao W, McCormick J, Cregan S, et al Dysregulated bioenergetics: a key regulator of joint inflammation. Ann Rheum Dis. 2016;75(12):2192–200. 10.1136/annrheumdis-2015-208476 27013493 PMC5136702

[14] McGarry T, Biniecka M, Gao W, Cluxton D, Canavan M, Wade S, et al Resolution of TLR2-induced inflammation through manipulation of metabolic pathways in Rheumatoid Arthritis. Scientific reports. 2017;7:43165 10.1038/srep43165 28225071 PMC5320554

[15] Jia W, Zhao X, Zhao L, Yan H, Li J, Yang H, et al Non-canonical roles of PFKFB3 in regulation of cell cycle through binding to CDK4. Oncogene. 2018;37(13):1685–98. 10.1038/s41388-017-0072-4 29335521

[16] Yalcin A, Clem BF, Simmons A, Lane A, Nelson K, Clem AL, et al Nuclear targeting of 6-phosphofructo-2-kinase (PFKFB3) increases proliferation via cyclin-dependent kinases. J Biol Chem. 2009;284(36):24223–32. 10.1074/jbc.M109.016816 19473963 PMC2782016

[17] Conradi LC, Brajic A, Cantelmo AR, Bouche A, Kalucka J, Pircher A, et al Tumor vessel disintegration by maximum tolerable PFKFB3 blockade. Angiogenesis. 2017;20(4):599–613. 10.1007/s10456-017-9573-6 28875379

[18] Jaffe EA, Nachman RL, Becker CG, Minick CR. Culture of human endothelial cells derived from umbilical veins. Identification by morphologic and immunologic criteria. The Journal of clinical investigation. 1973;52(11):2745–56. 10.1172/JCI107470 4355998 PMC302542

[19] Haraldsen G, Kvale D, Lien B, Farstad IN, Brandtzaeg P. Cytokine-regulated expression of E-selectin, intercellular adhesion molecule-1 (ICAM-1), and vascular cell adhesion molecule-1 (VCAM-1) in human microvascular endothelial cells. Journal of immunology (Baltimore, Md: 1950). 1996;156(7):2558–65. 8786319

[20] Israel A. The IKK complex, a central regulator of NF-kappaB activation. Cold Spring Harb Perspect Biol. 2010;2(3):a000158 10.1101/cshperspect.a000158 20300203 PMC2829958

[21] Li ZW, Chu W, Hu Y, Delhase M, Deerinck T, Ellisman M, et al The IKKbeta subunit of IkappaB kinase (IKK) is essential for nuclear factor kappaB activation and prevention of apoptosis. J Exp Med. 1999;189(11):1839–45. 10.1084/jem.189.11.1839 10359587 PMC2193082

[22] Dinarello CA. Biologic basis for interleukin-1 in disease. Blood. 1996;87(6):2095–147. 8630372

[23] Takaesu G, Ninomiya-Tsuji J, Kishida S, Li X, Stark GR, Matsumoto K. Interleukin-1 (IL-1) receptor-associated kinase leads to activation of TAK1 by inducing TAB2 translocation in the IL-1 signaling pathway. Mol Cell Biol. 2001;21(7):2475–84. 10.1128/MCB.21.7.2475-2484.2001 11259596 PMC86880

[24] Wang C, Deng L, Hong M, Akkaraju GR, Inoue J, Chen ZJ. TAK1 is a ubiquitin-dependent kinase of MKK and IKK. Nature. 2001;412(6844):346–51. 10.1038/35085597 11460167

[25] Mako V, Czucz J, Weiszhar Z, Herczenik E, Matko J, Prohaszka Z, et al Proinflammatory activation pattern of human umbilical vein endothelial cells induced by IL-1beta, TNF-alpha, and LPS. Cytometry A. 2010;77(10):962–70. 10.1002/cyto.a.20952 21290470

[26] Pollheimer J, Bodin J, Sundnes O, Edelmann RJ, Skanland SS, Sponheim J, et al Interleukin-33 drives a proinflammatory endothelial activation that selectively targets nonquiescent cells. Arterioscler Thromb Vasc Biol. 2013;33(2):e47–55. 10.1161/ATVBAHA.112.253427 23162017

[27] Madge LA, Pober JS. TNF signaling in vascular endothelial cells. Exp Mol Pathol. 2001;70(3):317–25. 10.1006/exmp.2001.2368 11418010

[28] Shi JH, Sun SC. Tumor Necrosis Factor Receptor-Associated Factor Regulation of Nuclear Factor kappaB and Mitogen-Activated Protein Kinase Pathways. Front Immunol. 2018;9:1849 10.3389/fimmu.2018.01849 30140268 PMC6094638

[29] Hart GW, Slawson C, Ramirez-Correa G, Lagerlof O. Cross talk between O-GlcNAcylation and phosphorylation: roles in signaling, transcription, and chronic disease. Annu Rev Biochem. 2011;80:825–58. 10.1146/annurev-biochem-060608-102511 21391816 PMC3294376

[30] Erickson JR, Pereira L, Wang L, Han G, Ferguson A, Dao K, et al Diabetic hyperglycaemia activates CaMKII and arrhythmias by O-linked glycosylation. Nature. 2013;502(7471):372–6. 10.1038/nature12537 24077098 PMC3801227

[31] Brooke DG, van Dam EM, Watts CK, Khoury A, Dziadek MA, Brooks H, et al Targeting the Warburg Effect in cancer; relationships for 2-arylpyridazinones as inhibitors of the key glycolytic enzyme 6-phosphofructo-2-kinase/2,6-bisphosphatase 3 (PFKFB3). Bioorg Med Chem. 2014;22(3):1029–39. 10.1016/j.bmc.2013.12.041 24398380

[32] Zhang R, Li R, Liu Y, Li L, Tang Y. The Glycolytic Enzyme PFKFB3 Controls TNF-alpha-Induced Endothelial Proinflammatory Responses. Inflammation. 2019;42(1):146–55. 10.1007/s10753-018-0880-x 30171427

[33] Edgell C, McDonald C, Graham J. Permanent cell line expressing human factor VIII-related antigen established by hybridization. Proceedings of the National Academy of Sciences. 1983;80(12):3734–3737. 10.1073/pnas.80.12.3734 PMC394125 6407019

