# Supplementary figures and images for: 3PO inhibits inflammatory NFκB and stress-activated kinase signaling in primary human endothelial cells independently of its target PFKFB3
Source: PLoS One. 2020 Mar 4;15(3):e0229395. doi: 10.1371/journal.pone.0229395 (PMC7055879; doi:10.1371/journal.pone.0229395)

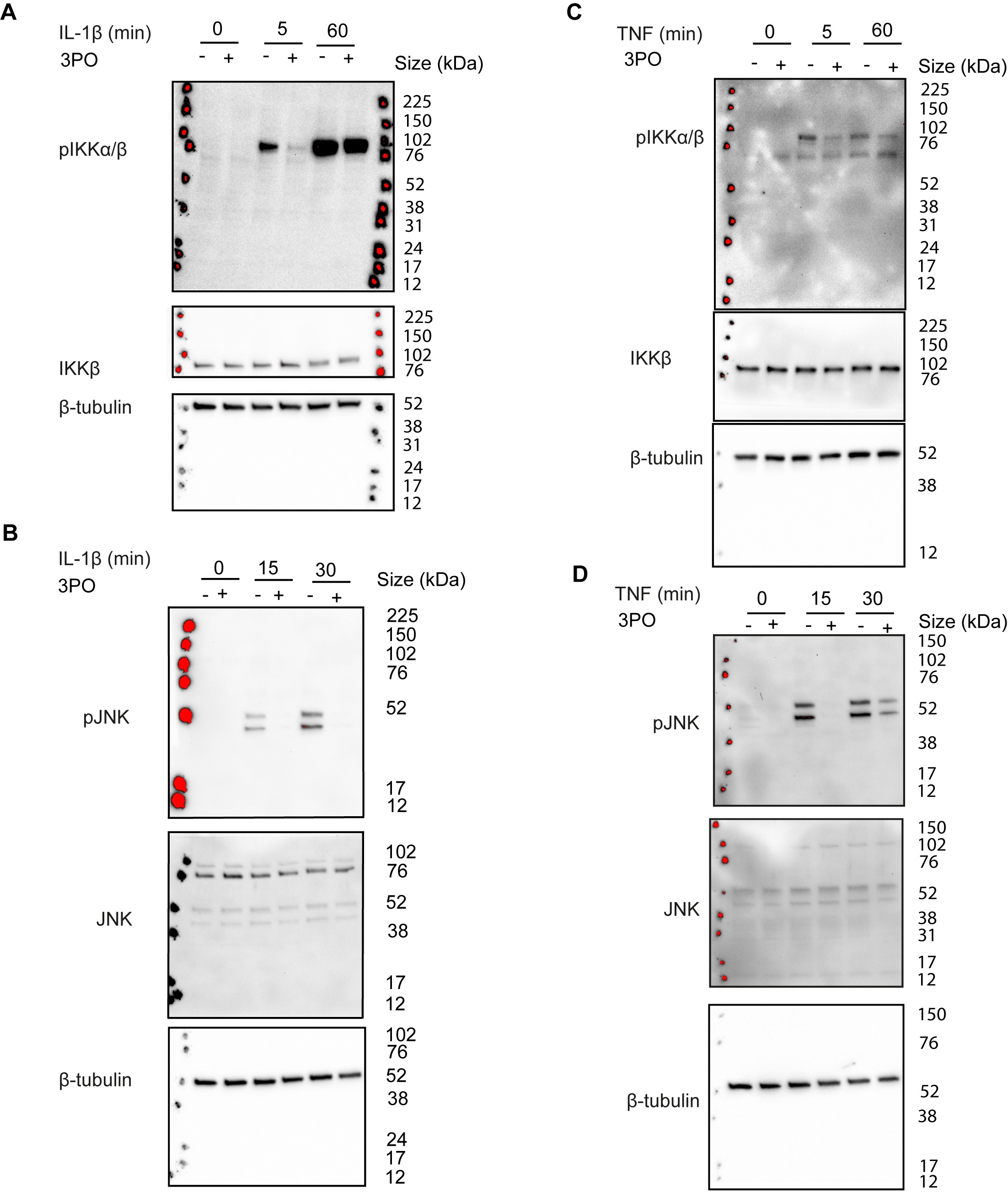

Supplement: S1 Fig — (A) Representative full-length immunoblots of HUVEC stimulated with IL-1β (1 ng/ml) for 0, 5, and 60 minutes in the presence (+) or absence of 3PO (20 μM) pretreatment for 30 minutes. Panels show phosphorylated IKKα/β (top) (mAb clone 16A6), total IKKβ (mAb clone D30C6) (middle), and β-tubulin loading control (rabbit polyclonal) (bottom). Two bands are visible on the membrane, with the lower band being unaffected by stimulation with IL-1β and most likely unspecific. After imaging of pIKKα/β, the membrane was stripped as detailed in materials and methods and cut between the 52 and 76 kDa marker before staining for total IKKβ and β-tubulin. (B) Representative full-length immunoblots of HUVECs treated as in panel A. Panels show phosphorylated JNK (top) (rabbit polyclonal), total JNK (middle) (rabbit polyclonal), and β-tubulin loading control (bottom) (rabbit polyclonal). The same membrane was used to stain for phosphorylated JNK, total JNK, and β-tubulin, in that order with stripping between staining. Two bands correspond to the estimated sizes of JNK (46 and 56 kDa) in addition to unspecific bands of higher molecular weight. (C) Representative full-length immunoblots of HUVEC stimulated with TNF (1 ng/ml) for 0, 5, and 60 minutes in the presence (+) and absence (-) of 3PO (20 μM) 30 minutes pretreatment. Panels show phosphorylated IKKα/β (top), total IKKα/β (middle), and β-tubulin loading control (bottom). Membranes were cut between the 52 and 76 kDa marker after imaging pIKKα/β. (D) Representative full-length immunoblots of HUVEC stimulated with TNF (1 ng/ml) for 0, 15, and 30 minutes in the presence (+) and absence (-) of 3PO (20 μM) pretreatment for 30 minutes Panels show phosphorylated JNK (top), total JNK (middle), and β-tubulin loading control (bottom). (TIF) [file pone.0229395.s001.tif]

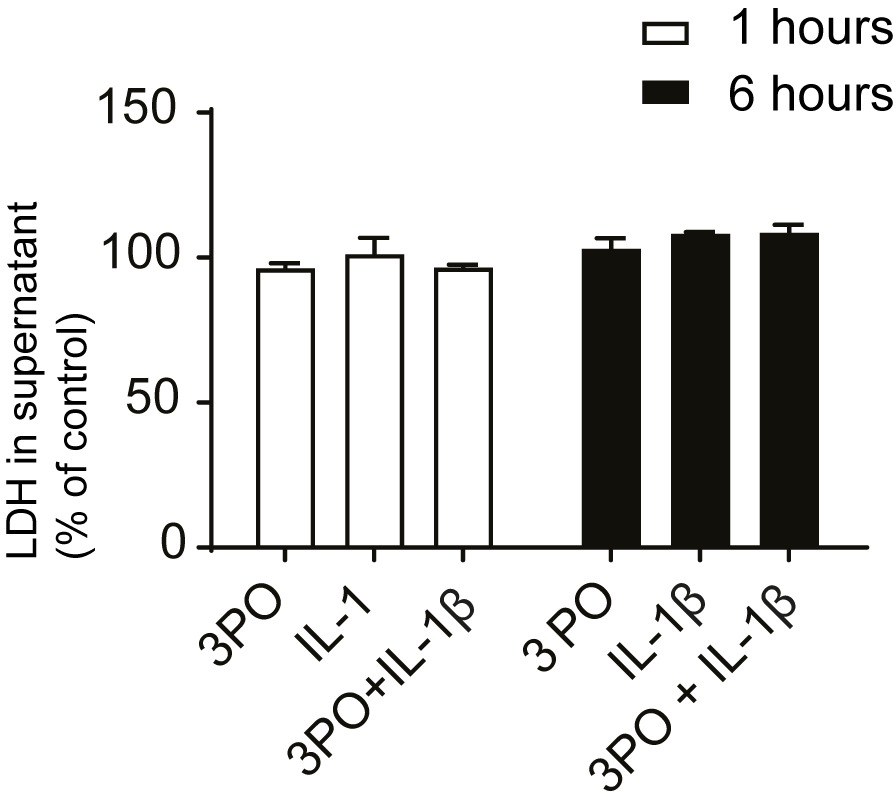

Supplement: S2 Fig — HUVECs were stimulated with IL-1β (1 ng/ml) in the presence or absence of 3PO (20 μM) for 1 or 6 hours. Supernatants were collected and toxicity was measured using the Cytotoxicity Detection KitPLUS (LDH) (Sigma 4744926001) according to the manufacturer’s instructions. LDH levels were normalized to control (n = 2). (TIF) [file pone.0229395.s002.tif]

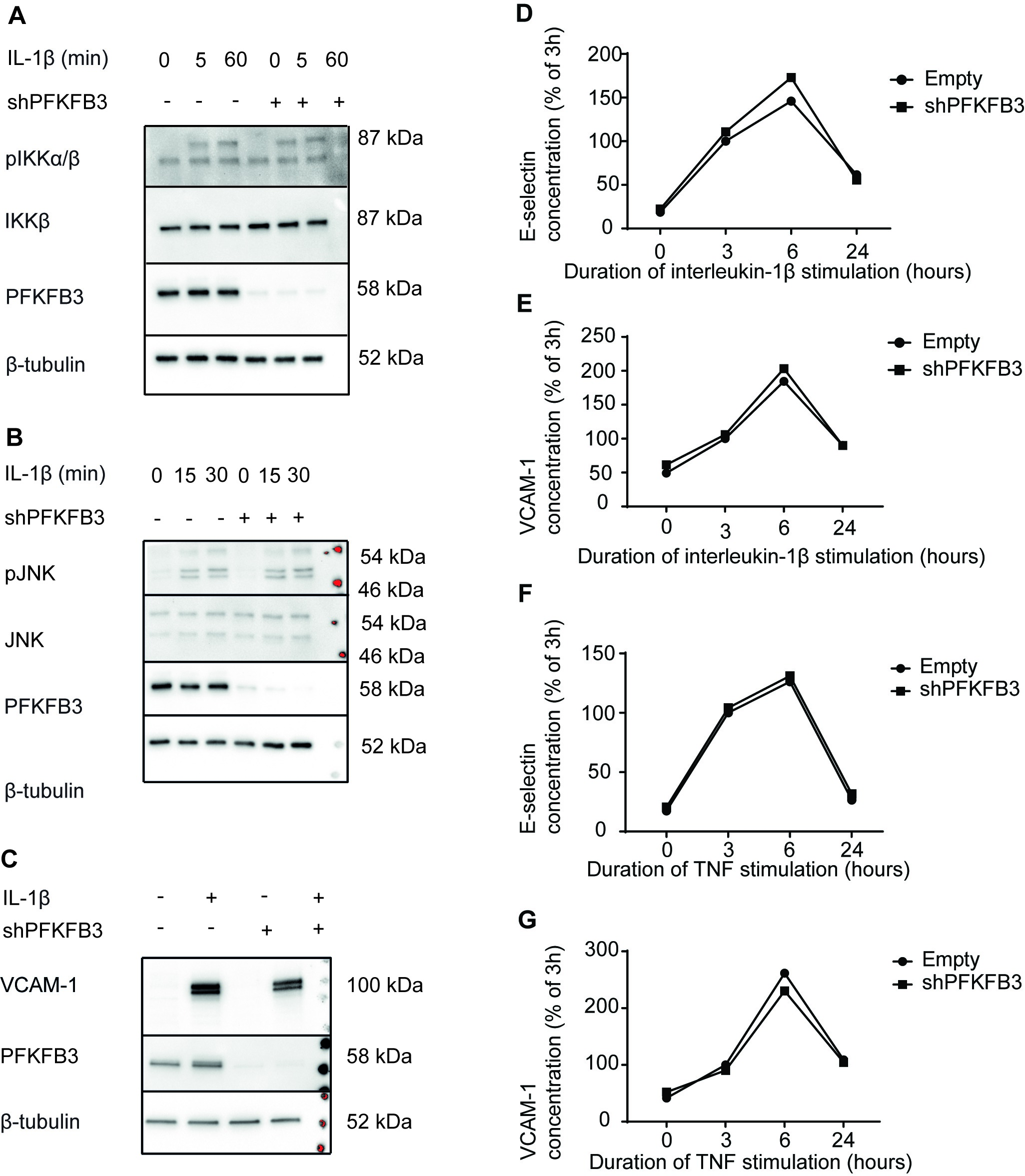

Supplement: S3 Fig — Representative blots (n = 2) of HUVECs transduced with lentivirus without shRNA (Empty) or lentivirus expressing shRNA against PFKFB3 (shPFKFB3) then stimulated with IL-1β for 5 and 60 minutes for phosphorylation IKKα/β (A), 15 or 30 minutes for phosphorylation of JNK (B) or 3 hours for protein level of VCAM-1 (C). A: Panels show phosphorylated IKKα/β (top) (mAb clone 16A6), total IKKβ (mAb clone D30C6) (middle), and β-tubulin loading control (rabbit polyclonal) (bottom) B: Panels show phosphorylated JNK (top) (rabbit polyclonal), total JNK (middle) (rabbit polyclonal), and β-tubulin loading control (bottom) (rabbit polyclonal). C: Panels show VCAM-1 (top) (polyclonal goat) β-tubulin (bottom) (polyclonal rabbit). Representative cell ELISA experiment (n = 2) showing the IL-1β-induced (1 ng/ml) upregulation of E-selectin (D) or VCAM-1 (E) or TNF-induced (1 ng/ml) upregulation of E-selectin (F) or VCAM-1 (G) after 3, 6 and 24 hours of stimulation in control cells (Empty) and in PFKFB3 knockdown cells (shPFKFB3). (TIF) [file pone.0229395.s003.tif]

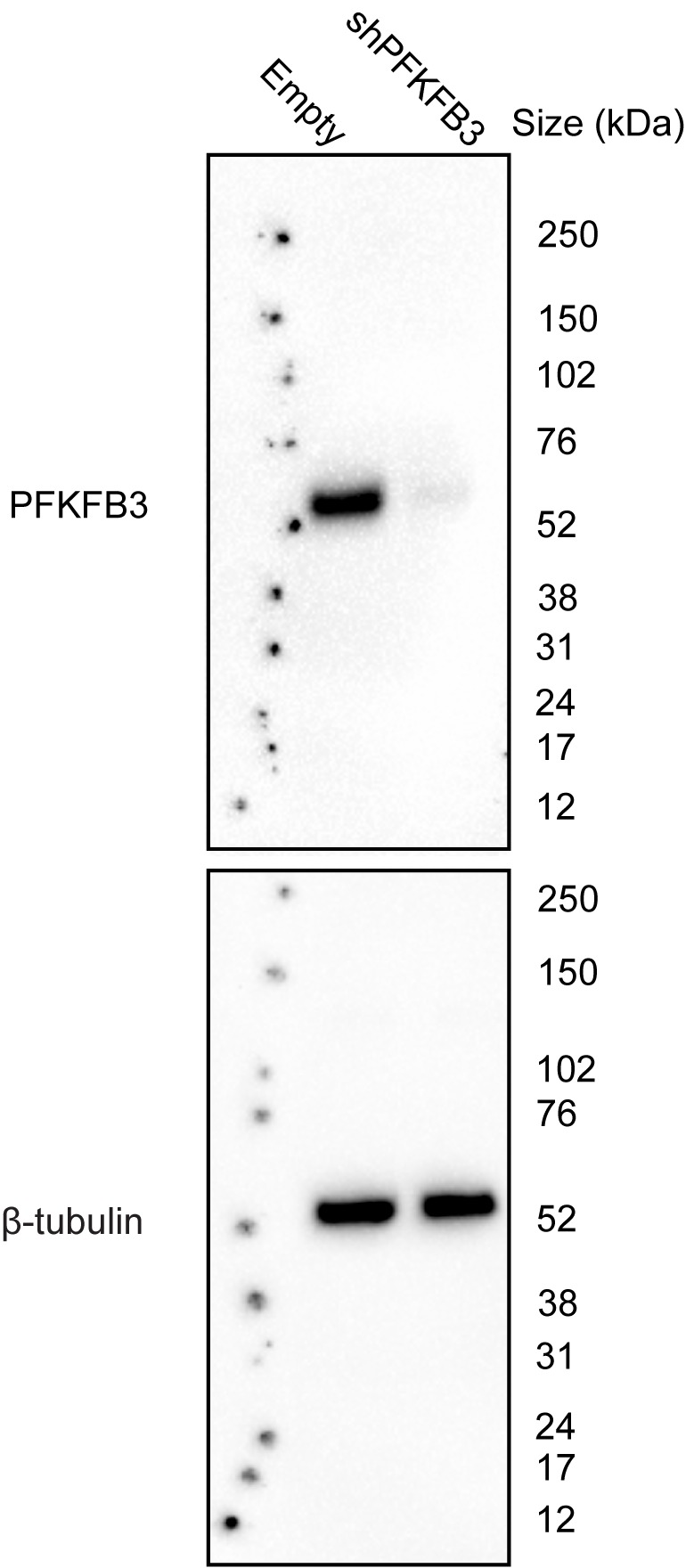

Supplement: S4 Fig — Representative full-length immunoblot of HUVECs transduced with lentivirus without shRNA (Empty) or lentivirus expressing shRNA against PFKFB3 (mAb clone EPR12594) (shPFKFB3). β-tubulin (rabbit polyclonal) was used as loading control. The membrane was stripped after imaging PFKFB3 and stained for β-tubulin. (TIF) [file pone.0229395.s004.tif]

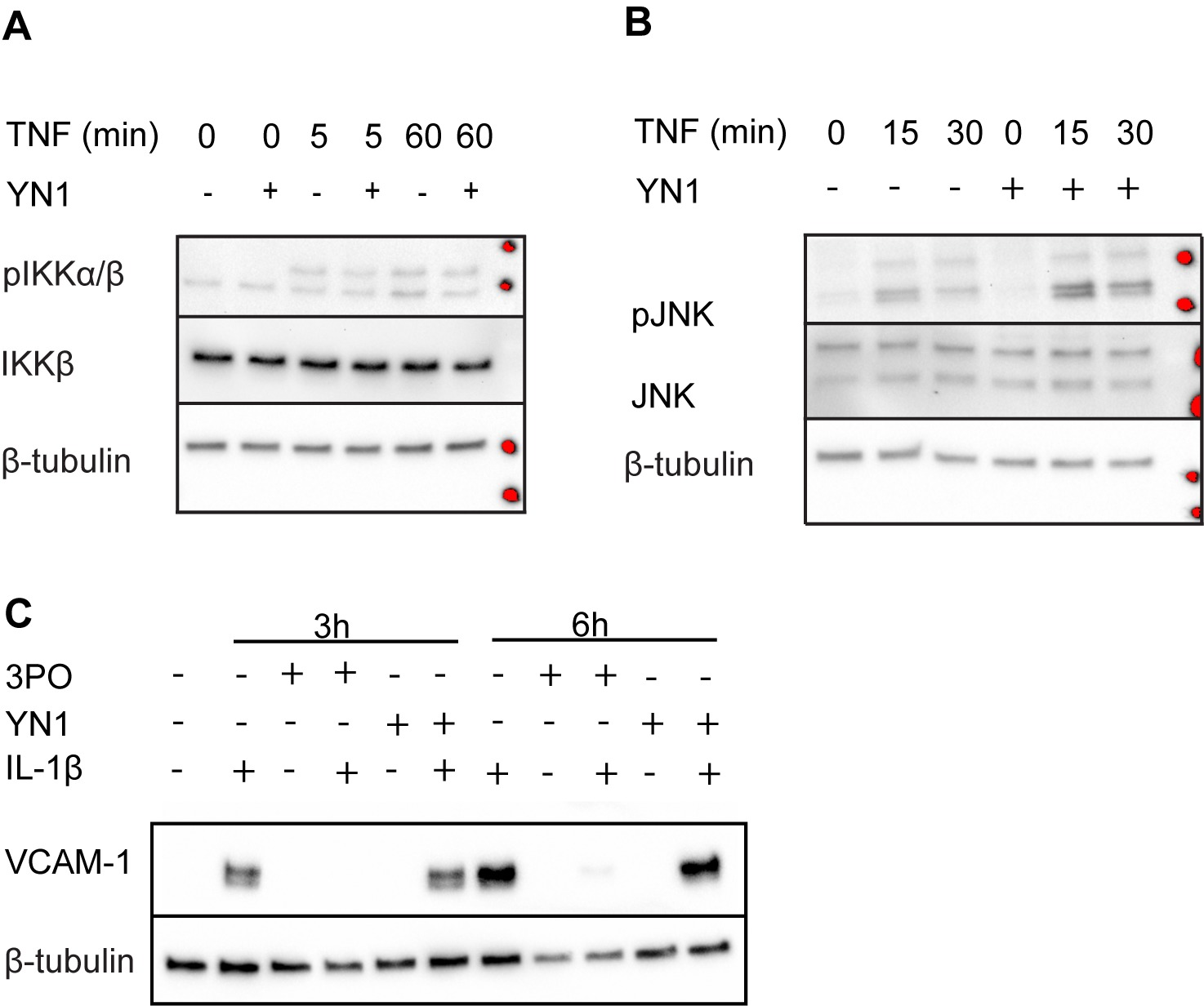

Supplement: S5 Fig — A: Representative immunoblots of cell extracts from HUVECs stimulated with IL-1β (1 ng/ml) for 0, 5 or 60 minutes after pretreatment for 30 minutes with medium or YN1 (20 μM). Panels show phosphorylated IKKα/β (top), total IKKβ (middle), and β-tubulin loading control (bottom). B: Representative immunoblot (n = 2) of cell extracts from HUVECs stimulated with IL-1β (1 ng/ml) for 0, 15 or 30 minutes after pretreatment for 30 minutes with medium or YN1 (20 μM). Panels show phosphorylated JNK (top) (rabbit polyclonal), total JNK (middle) (rabbit polyclonal), and β-tubulin loading control (bottom) (rabbit polyclonal). C: Representative immunoblot (n = 2) of cell extracts from HUVECs stimulated with IL-1β (1 ng/ml) in the presence or absence of 3PO (20 μM) or YN1 (20 μM) for 3 or 6 hours. Panels show VCAM-1 (top) (polyclonal goat) β-tubulin (bottom) (rabbit polyclonal). (TIF) [file pone.0229395.s005.tif]

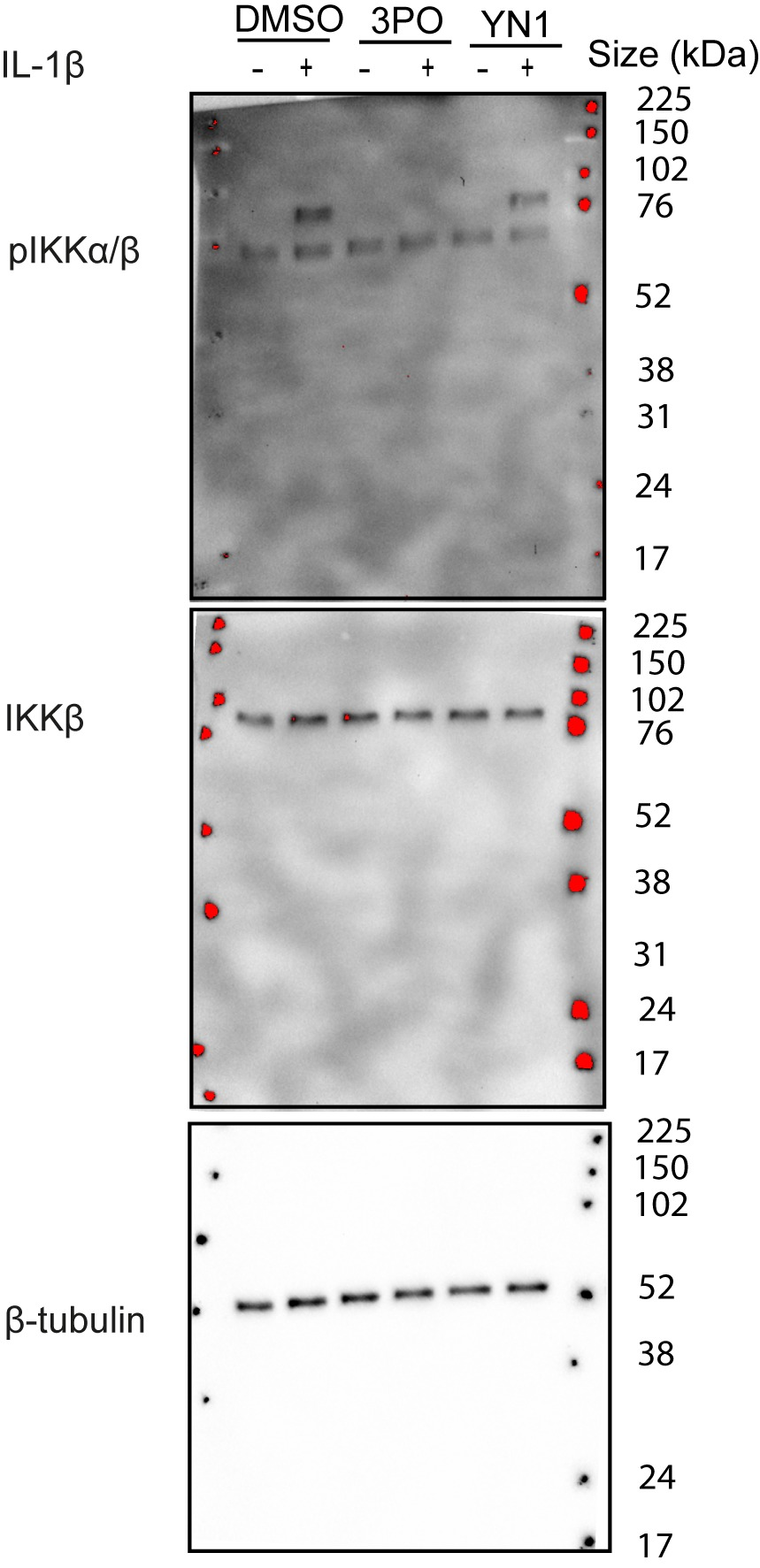

Supplement: S6 Fig — Representative full-length immunoblots of cell extracts from HUVECs stimulated with IL-1β (1 ng/ml) for 0 (-) or 5 (+) minutes without (-) or with (+) pretreatment with either 3PO (20 μM), or YN1 (20 μM) for 30 minutes. Panels show phosphorylated IKKα/β (top) (mAb clone 16A6), total IKKβ (mAb clone D30C6) (middle), and β-tubulin loading control (rabbit polyclonal) loading control (bottom). There is a band of slightly smaller molecular weight than expected visible in all lanes for pIKKα/β that seems to be unaffected by either IL-1β stimulation or treatment with 3PO or YN1 and is likely unspecific. The same membrane was used to stain for phosphorylated IKKα/β, total IKKβ, and β-tubulin, in that order with stripping between staining. (TIF) [file pone.0229395.s006.tif]

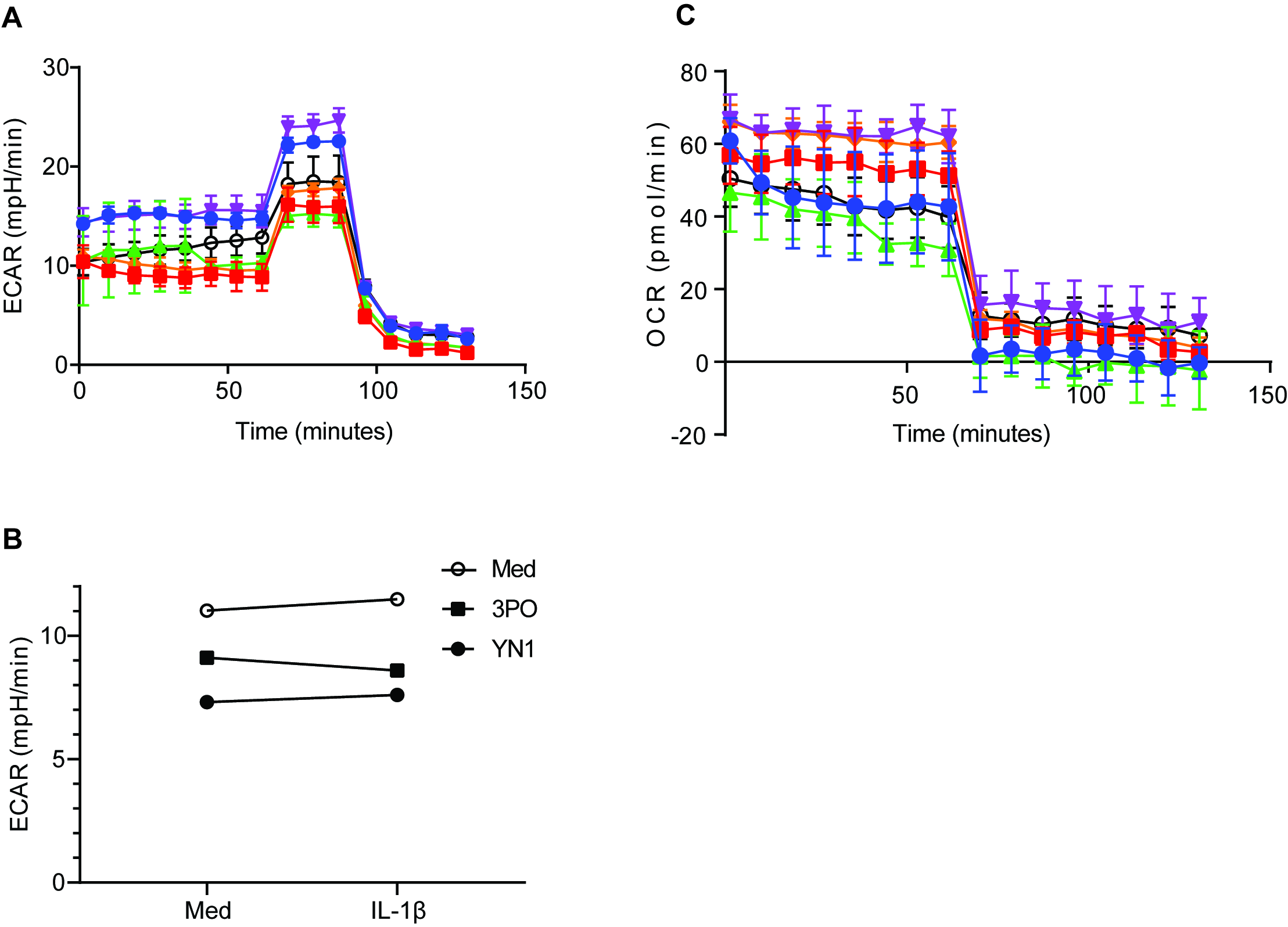

Supplement: S7 Fig — Representative (n = 2) seahorse experiment showing untreated HUVECs (untreated) or treated with 3PO (20 μM) or YN1 (20 μM) for 1 hour. IL-1β or DMEM (med) is injected at 1, rotenone/antimycin A at 2 and 2-deoxyglucose at 3 then ECAR (A) and ECAR was compared in in cells stimulated with IL-1β compared to non-stimulated cells (B) and OCR was meassured. Representative of 2 independent experiments. (TIF) [file pone.0229395.s007.tif]
